# Improving of Type 2 Diabetic Patients’ Knowledge, Attitude and Practice Towards Diabetes Self-care by Implementing Community-Based Interactive Approach-Diabetes Mellitus Strategy

**DOI:** 10.1186/1756-0500-5-315

**Published:** 2012-06-21

**Authors:** Titien Siwi Hartayu, Mohamed Izham MI, Sri Suryawati

**Affiliations:** 1 Faculty of Pharmacy Sanata Dharma University, Yogyakarta, Indonesia; 2 College of Pharmacy, Qatar University, Doha, Qatar; 3 Department of Pharmacology and Therapy, Faculty of Medicines, Gadjah Mada University, Yogyakarta, Indonesia

**Keywords:** CBIA-DM, Diabetes mellitus, KAP, Public education, Small group discussion

## Abstract

**Background:**

Community Based Interactive Approach-diabetes mellitus (CBIA-DM) is an active self-learning method. This study is aimed at improving type 2 diabetic patients' knowledge, attitude and practice on diabetes self-care by implementing the CBIA-DM strategy. Time series, pre and post quasi-experimental design, Intervention group underwent CBIA-DM, DM-club and Normal-care group acted as control. Data were collected in pre-intervention, immediately, one, three and six months post intervention. Ranging scores for pre and post test questionnaires were: knowledge (0–18) and attitude (9–45); categorizing as rational scales of the scores in good, fair and poor. Practicing in diabetes self-care was assessed using 12 questionnaires, and categorized as adhere and not adhere to DM self-care. Effectiveness of CBIA-DM was evaluated based on the increasing number of participants in good knowledge and attitude levels, and adherence in practicing diabetes self-care.

**Results:**

CBIA-DM group shows increasing number of participants in good level of knowledge from 40 % (n = 30) up to 80 % at M + 3 with scores significantly improved from 13.1 ± 2.4 up to 15.4 ± 2.0 (Wilcoxon test, p < 0.05), attitude from 20 % up to 50 % at M + 3, with scores significantly improved from 33.5 ± 4.1 up to 34.9 ± 6.2 (p = 0.031) and increasing number of participants’ adherence to all variables of DM self-care at M + 6 post intervention.

**Conclusions:**

CBIA-DM strategy is effective to improve diabetic patients’ knowledge, attitude and practice on diabetes self-care. Repeating and improving the strategy program is needed to sustain the impact.

## Background

Community Based Interactive Approach (CBIA) is a method used for public education which emphasized on the active role of participants in looking for information. The CBIA was first developed by Suryawati in 1993 as Mothers Active Learning Method to improve knowledge and skills in selecting OTC medicines using inserts package of OTC medicines as training material, impacted in reducing OTC medicines in household. The intention of CBIA is to empower participant to seek and critically assess information about their treatment [1]. The CBIA has been proven effective to improve knowledge and skills of pharmacy assistant in hypertension drug information service in Yogyakarta by Astuti (1998) [2]. The CBIA has also succeeded to increase “the level of knowledge and improving skills in early detection of breast cancer by Sunarsih (2002)”, and then improving patients’ adherence to treatment program in Tuberculosis [3]. CBIA was not only targeted to the patient but also to the patients’ family and care giver. Targeted patients’ family and care giver could be expected to motivate not only the patient but also their friends or colleague to improve patients’ adherence to treatment program through sharing knowledge and experiences.

Diabetes mellitus (DM) is a group of metabolic diseases which is characterized by the high levels of blood glucose resulted from defects in insulin secretion, insulin action, or both [4]. The incidence of Type 2 diabetes is increasing worldwide [5] and in a global scale, DM is one of the top five diseases which cause death [6]. Diabetes mellitus affected almost 150 million people worldwide, and in the year of 2025, the number of diabetic people is estimated to increase until 300 million [7]. Meanwhile, other researchers in other studies estimated that in the year 2030 the number of diabetics will rise up to 366 million people from 171 million people in 2000 [8] and it is predicted that the developing countries have their contribution which those 70 % of diabetic people are living there [9,10]. Indonesia is one of developing countries, where the prevalence of diabetes mellitus is also increasing rapidly. In Indonesia, it has another estimation of the number of diabetes people, with total population of 125 million in 2000, which will be increasing in the next 30 years that the diabetes people would be 21.3 million from 8.4 million in 2000, meaning that it is increasing 3 times [11]. It can be understood that it would not be enough health professionals which are available in Indonesia to manage this problem. This study was aimed at improving diabetic patients’ knowledge, attitude and practice in diabetes self-care by implementing CBIA-DM strategy.

## Methods

This is a time series pre and post quasi-experimental with control groups design [12]. The population of the intervention group are the community member of the Sanata Dharma University who suffered from Type 2 diabetes (87 persons), whilst the control groups are member of charity hospital DM-club and diabetic patients of public hospital. The participants are selected among the population based on the inclusion criteria, i.e. male or female with Type 2 diabetes mellitus, literate, had not attended a diabetes education program in the last 2 years to minimize confounding factors, consented to the program and completed all activities required over the six months period. Among 87 persons of the population, there are only 30 persons that fulfill the inclusion criteria as intervention group. Therefore, the total amount of the control groups were corresponded with the intervention group, i.e. 30 person with the same inclusion criteria in each group. The intervention group underwent CBIA-DM strategy. The control groups are: One group as DM club group, they do Sundays meeting for physical activity together and 2 monthly regularly seminars, and one group received normal care. Based on ethics of study and the activities program that patients have to follow, patients who are pregnant, walking are contraindicated (e.g. renal failure or previous amputation), and mentally handicapped are excluded in this study.

All participants underwent pre test before conducting CBIA-DM strategy and post test at immediately, one month, three months and six months post intervention. The intervention is conducted in a form of small-group problem-based intensive discussions followed by individual self-assessment. Members of each small-group are six persons, and five small groups involved in this study. Senior Pharmacy students are recruited as discussion facilitators. The facilitators show the way to find or solve the problem if necessary during discussion. Before carrying out the CBIA-DM, facilitators underwent a short training by the investigator to familiarize them with diabetes problem. Internists and nutritionist are responsible for responding to scientific problems found during the discussion. A CBIA-DM package is used as an educational material; consisting 7 booklets as follows: Booklet 1: Activities guide, Booklet 2: (issues of DM), Booklet 3: About DM, Booklet 4: Healthy life style, Booklet 5: Exercises, Booklet 6: Foot care, Booklet 7: Diet program. The booklets contained information about diabetes management and healthy life style for people with diabetes. The booklets are developed based on diabetic patients’ problems, using focus group discussion (FGD) among the target community [13]. CBIA-DM package is tested by lay people for the language whether it is easy to understand and do not create misconception. Lay outing contents of the CBIA-DM package is reviewed by internist, nutritionist and head of diabetic club as well as its layout. Comments and advices incorporated in the booklets revision. The CBIA-DM program takes two hours. Investigator begin with a prologue on the advantages/disadvantages of adherence to the treatment program, and then participants are requested to form small-groups. Group leaders (elected from each group) and facilitators conduct the CBIA program using CBIA-DM packages and worksheets.

Pre and post test KAP questionnaires are used as study instruments. Participants’ answers in the knowledge questionnaires are scored, correct answer is 2, incorrect answer is 0 and doubtful answer is 1. Attitude questions for favourable questions are scored as follows: 5 for strongly agree, 4 for agree, 3 for doubtful, 2 for disagree, and 1for strongly disagrees. In unfavourable questions, the scores are 5 for strongly disagree, 4 for disagree, 3 for doubtful, 2 for agree, and 1for strongly agree. Therefore, knowledge score range from 0–18 and attitude score range from 9–45. Each score is categorized as rational scales in good (> 14), fair (12–14) and poor (< 12) for knowledge levels, and good (> 35), fair (30–35) and poor (< 30) for attitude levels [14]. Practicing in diabetes self-care is assessed using 12 questions, and then categorized as adhere and not adhere on diabetes self-care. The knowledge test of the participants are out of the total number of specific groups participants (CBIA-DM group, DM club group, Normal care group). Effectiveness of CBIA-DM is evaluated based on the increasing number of participants in good knowledge and attitude levels, and adhere to practice diabetes self-care. The summary results are presented in percentage of participants who are in a good, fair or poor level [15]. Comparisons of diabetic patients’ KAP pre and post intervention for each group are analyzed using Wilcoxon signed-rank test, and comparisons between groups are analyzed using Mann–Whitney test, p < 0.05 is considered statistically significant.

The ethical clearance of this study was obtained from the ethical committee of research in Medical Health Faculty of Medicine Gadjah Mada University, Yogyakarta, Indonesia (Ref: KE/MK/573/EC) and was supported by participants’ inform consent as agreement to attend all of the activities required during study voluntarily.

## Results

The highest amount of participants in the study was female in the CBIA-DM group (57 %, n = 30), with mean of age 55.3 years (ranges 39 – 68). Participants’ education levels were equal in the three groups as the most appropriately state to compare the impact of strategy intervention among the three groups. Relative to duration of illness, CBIA-DM group (n = 30) was less than 5 years (23 %) equal with normal care group, while DM club was the highest among the three groups (43 %). The highest usage of medicines in the three groups was metformin which reached up to 63 % in normal care group, 60 % in the CBIA-DM group and 59 % in DM club. The summary results is showed in the Table 1.

**Table 1:** Demographic characteristics of participants problem with alignment

**No**	**Characteristics of participants**	**CBIA-DM**		**Normal care**		**DM-Club**	
		**n**	**%**	**n**	**%**	**n**	**%**
**1**	**Gender**						
	− Male	13	43	10	33	12	40
	− Female	17	57	20	67	18	60
**2**	**Average age (years old)**	55.3 (39 – 68)		56.4 (39–67)		58.3 (41–75)	
**3**	**Level of education**						
	− Elementary school	4	13	4	14	3	10
	− High school	14	47	16	53	16	53
	− University	12	40	10	33	11	37
**4**	**Employment**						
	− Employee	9	30	22	74	4	13
	− Employer	9	30	1	3	5	17
	− Unemployed	10	33	7	23	11	37
	− Pensioner	2	7	-	-	10	33
**5**	**Marital status**						
	− Single	2	7	2	7	1	3
	− Married	25	83	23	76	24	80
	− Widow/widower	3	10	5	17	5	17
**6**	**Duration of illness**						
	− < 5 years	7	23	7	23	13	43
	− 5 – 10 years	15	50	16	53	10	33
	− 11 – 16 years	5	17	5	17	5	17
	− > 16 years	3	10	2	7	2	7

**Figure 1:**
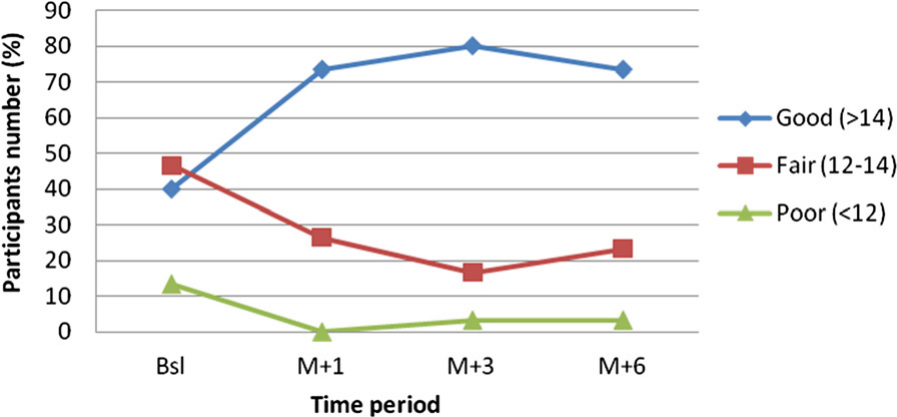
Distribution of CBIA-DM participants in knowledge levels.

Number of participants in good knowledge level of CBIA-DM group significantly increased from 40 % (n = 30) up to 73.4 % and reached a peak in 80 % with scores improved from 13.1 ± 2.4 up to 15.4 ± 2.0 (Wilcoxon test, p < 0.05). On the other hand, DM club increased from 53.8 % (n = 30) up to 86.7 % (Wilcoxon test, p = 0.012), while normal care showed decreasing trend from 36.6 % (n = 30) at baseline into 23.3 % (Wilcoxon test, p = 0.080). Decreasing number of participants in poor level was achieved by CBIA-DM and DM club at M + 1 (0 %) from 13.4 % and 6.6 %, respectively. Unfortunately, both in CBIA-DM and DM club at M + 3 and M + 6 poor level rose up to 3.3 %, while normal care rose up from 6.6 % to 13.4 %. (The summary result is showed in Table 2 and Figure 1).

**Table 2:** **Distribution of participants in the level of knowledge (score 0–18): Good (= > 14); Fair (= 12–14); Poor (= < 12) (Badrudin*ET AL*., 2002)**

	**Normal Care (n = 30)**				**CBIA-DM (n = 30)**				**DM-Club (n = 30)**			
**Time Period/ Levels**	Bsl*	**M + 1**	**M + 3**	**M + 6**	**Bsl**	M + 1*	M + 3*	M + 6*	**Bsl**	**M + 1**	**M + 3**	**M + 6**
**Good (%)**	36.6	NA**	20.0	23.3	40.0	73.4	80.0	73.4	53.8	70.0	66.7	86.7
**Fair (%)**	56.8	NA	73.4	63.3	46.6	26.6	16.7	23.3	40.0	30.0	30.0	10.0
**Poor (%)**	6.6	NA	6.6	13.4	13.4	0.0	3.3	3.3	6.6	0.0	3.3	3.3

Bsl:Baseline; M + 1: one month; M + 3: three months; M + 6: six months post intervention. NA: Not available; *Wilcoxon test, p < 0.001.10.

**Figure 2:**
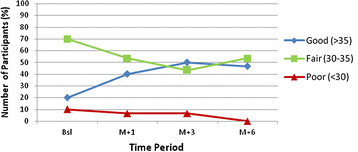
Distribution of CBIA-DM participants in attitude towards diabetes self-care.

Good level in attitude toward diabetes and diabetes management of CBIA-DM group increased from 20 % up to 46.6 % and reached a peak in 50 % with scores significantly improved from 33.5 ± 4.1 up to 34.9 ± 6.2 (Wilcoxon test, p = 0.031). On the other hand, DM club and normal care were also improved, but the improvement were not statistically significant (Wilcoxon signed rank test, p = 0.110 and p = 0.082, respectively). (The summary results in Table 3 and Figure 2).

**Table 3:** **Distribution of participants in the level of attitude (score 9–45) towards diabetes self-care: Good(= > 35); Fair (= 30–35); Poor (= < 30) (Badrudin*et al*., 2002)**

	**Normal Care (n = 30)**				**CBIA-DM (n = 30)**				**DM-Club (n = 30)**			
**Levels Period/Levels**	**Bsl**	**M + 1**	**M + 3**	**M + 6**	**Bsl**	M + 1*	M + 3*	M + 6*	**Bsl**	**M + 1**	**M + 3**	**M + 6**
**Good (%)**	18.5	NA	26.6	26.6	20	40	50	46.6	30	33.4	33.4	36.7
**Fair (%)**	76.6	NA	73.4	73.4	70	53.4	43.4	53.4	60	60	56.6	60.0
**Poor (%)**	6.6	NA	0	0	10	6.6	6.6	0	10	6.6	10	3.3

Baseline; M + 1: one month; M + 3: three months; M + 6: six months post intervention NA: Not available; *Wilcoxon test, p = 0.031.

Practising on diabetes self-care is showed by the number of CBIA-DM participants’ adherence to all variables of diabetes self-care. Results of the study showed the increasing number of CBIA-DM participants who adhere to all diabetes self-care variables. On the other hand, Normal care group shows steady stage in not-adhere to almost all variables of diabetes self-care (Table 4).

**Table 4:** Distribution of CBIA-DM participants who adhere on practicing DM self-care variables

	**Normal care group (n = 30)**		**CBIA-DM group (n = 30)**	
**DM Self-care Variables**	**Baseline (%)**	**M + 6 (%)**	**Baseline (%)**	**M + 6 (%)**
Blood Pressure test	61.0	61.0	93.3	100
Vision test	24.4	24.4	26.7	50
Blood glucose level test	46.3	50.0	43.3	70
Regular exercises	63.4	50.0	70	100
Diet program	61.0	61.0	63.4	90
Taking medication	56.1	46.1	62.7	90
Foot care	24.4	40.0	30	100

## Discussion

The majority of participants in all groups are females with the age is 39 years and over and duration of illness in all groups are equal. This indicates that this problem might be more prevalent among females than males [16-18]. The youngest participant is 39 years much younger than in European population [10] which the youngest of diabetic patient is 50 years old (ranges 50–60 years) and also younger than it is characterized according to Soegondo (2007) [19] which is 45 years. Participants’ education levels are equal in the three groups as the most appropriate statement to compare the impact of the intervention strategy among the three groups [14,20]. Most of study participants (> 75 %) in the three groups are married, this situation is expected that their family can support and motivate participants in doing the diabetes self-care which will lead in increasing patients’ adherence to treatment program.

Before conducting the study, patients’ education has regularly been given in DM club through Sunday’s meeting and 2 monthly seminars. Therefore, among the three groups, the highest amount of participants who have good level of knowledge at baseline is DM club, while CBIA-DM group and normal care group are equal due to both groups are not receive any patient education about diabetes except from the physician when they visit for medical check up. However, during the study period, numbers of participants who have a good level of knowledge in normal care are decreasing. On the other hand, CBIA-DM group and DM club increase, even though CBIA-DM slightly decreases at M + 6. Comparing the trend of increasing knowledge in CBIA-DM group and DM club, CBIA-DM is better than DM club, because CBIA-DM group only receives once diabetes education resulted in increasing participants’ knowledge attitude and skill in diabetes self-care, which steadily remains during the 6 months study period. On the other hand, DM club receives diabetes education regularly, and more often than CBIA-DM group results in fluctuated trend. Improving knowledge of CBIA-DM participants is showed by the significant difference between knowledge score in baseline and immediately (Wilcoxon test, p < 0.001), between baseline and M + 1 (Wilcoxon test, p < 0.001), between baseline and M + 3 (Wilcoxon test, p < 0.001) and between baseline and M + 6 (Wilcoxon test, p < 0.001). These results indicated that CBIA-DM is effective in improving knowledge of participants which is supported by the previous study that knowledge can be improved through training and education, and educational model with involving an active role of the participants result in improving knowledge significantly and steadily as based for behaviour changing [21,22]. However, due to the slightly decrease at M + 6, repeating education before M + 6 follow up intervention and improving the program might be worthy to sustain the high knowledge level.

Related to the attitude and practice level, the study shows the increasing numbers of participants who have good level of attitude and practice towards diabetes and diabetes self-care in all groups, with CBIA-DM group achieved the highest number of participants. Attitudes and practice are influenced by cultural and religious teachings, as well as school, the peer group, parents, and life experience [23]. According to the Triadic schema attitude has 3 components which support each other, e.g. cognitive component, affective component, and co-native component. Cognitive component is the representation of what they believed, while affective component is the emotional aspect in feeling and co-native component is the tendency to have certain behaviour based on the attitude [24]. Intervention using CBIA-DM is conducted through interactive small group discussion that led the participants to involve each other through sharing their experience and information. In addition, considering the intention of CBIA strategy is to empowering participants seeking and critically assessing information about their treatment that will lead to motivate participants in changing behaviour which is resulted by learning [1]. Moreover, the most important which supported the success of education is the educational material (CBIA-DM package) in conducting CBIA-DM which was developed based on the real of participants’ needs explored using Focus Group Discussion among the aspirants. Thereby, even though only once intervention, CBIA-DM is proven increases attitude level towards diabetes self-care. Unlike in DM club group, which diabetes educations are given through 2 monthly regular seminars and Sundays’ meeting, more often than CBIA-DM, however the increasing attitude is not as sharp as CBIA-DM. However, DM club is of course higher than Normal Care.

Based on the study results and it is supported by the findings in “A pilot implementation study in private maternity hospital that CBIA strategy improved skill in selecting OTC medicines for common cold in pregnancy” by Hidayati et al., (2009) and “improving skill in early detection of breast-cancer” by Sunarsih (2002), therefore CBIA-DM is unavoidable as an effective strategy in improving skill on diabetes self-care. In addition, number of CBIA-DM participants who are in a good level both of knowledge, attitude and practice level, which sustain until 6 months after conducting the intervention indicates the effectiveness of CBIA-DM in improving diabetic patients in managing healthy lifestyle for people with DM. CBIA-DM group are only receive once education during 6 months study period, while DM club group has Sundays meeting which led more frequently and intensively in sharing experiences among the members beside receive more educations. However, when the results are compared to DM club group, some results of CBIA-DM group are better.

Difficulties in motivating the participants to follow and to complete all activities required during the study even though they have signed the informed consent forms lead to low sample size of study participants, which restricted the analysis for many variables. Moreover, with the small sample size the authors are not certain whether generalization can be made and extrapolation of the results to the general population is questionable.

## Conclusions

CBIA-DM strategy is effective in improving type 2 diabetic patients’ knowledge, attitude, and practice towards diabetes self-care. Improving and repeating the program in 6 months post intervention is needed to sustain the good level.

## Competing interests

The authors declare, there is no conflict of interest in financial either directly or indirectly associated with this manuscript.

## Authors’ contributions

All authors contributed to design of the study. TSH carried out data collection, data analysis, data interpretation and drafted the manuscript. MIMI and SS clarified the data and revised the manuscript. All authors read and approved the submitted manuscript.

## References

[B1] Suryawati S CBIA: Improving the quality of self-medication through mothers’ active learning Essential Drugs Monitor. World Health Organization. Geneva 2003 32 22 23

[B2] Astuti A Improving knowledge and skills of pharmacy assistances in Yogyakarta municipality on medicine information service for hypertensive patients using community based interactive approach (CBIA) Master Thesis Gadjah Mada University, Master program for medicine management and policy studies 1998

[B3] Susantinimplementing CBIA strategy to improve tuberculosis patients’ adherence to treatment program. Master Thesis. Gadjah Mada University, Master program for medicine management and policy studie 2006,

[B4] Suyono S Latest Guidelines in Management of Type 2 Diabetes Mellitus Diabetes Seminar 2009 Jakarta

[B5] Kuller LH Dietary fat and chronic diseases epidemiologyc: Overview J Am Diet Assoc 1997 97 9 15 10.1016/s0002-8223(97)00724-4 9216562

[B6] Robles YHK Edwards AGK Cannings-John R Butler C Health education for type 2 diabetes mellitus in ethnic minority groups (protocol) In The Cochrane Library 2007 2 1 16

[B7] Adherence to long-term therapies: evidence for action 2003 World Health Organization, Switzerland

[B8] Wild S Roglic G Green A Sicree R King H Global prevalence of diabetes: estimates for the year 2000 and projections for 2030 Diabetes care 2004 27 Suppl 5 1047 53 15111519 10.2337/diacare.27.5.1047

[B9] Ebrahim S Davey SG Exporting failure coronary heart disease and stroke in developing countries Int. J Epidemiol 2007 30 201 5 11369713 10.1093/ije/30.2.201

[B10] Ghazanfari Z Ghofranipour F Tavafian SS Ahmadi F Rajab A Life style education and diabetes mellitus type 2: A non-randomized control trial Iranian Journal Public Health 2007 36 Suppl 2 68 72

[B11] Sutanegara D Budiarta The epidemiology and management of diabetes mellitus in Indonesia 2000 [http://www.ncbi.nlm.nih.gov/entrez/query.fcgl?cmd=retrieve&db] 10.1016/s0168-8227(00)00173-x 11024578

[B12] Abramson JH Survey methods in community medicine 1990 4 Edinburgh, Churchill Livingstonel 399

[B13] Grbich C Qualitative research in health: an introduction 1999 Sage Publications, London 222 227

[B14] Badrudin N Basit A Hydrie MZI Hakeem R Knowledge, attitude and practices of patients visiting a diabetes care unit Pakistan Journal of Nutrition 2002 1 99 102

[B15] Issa BA Baigewu O Quality of life of patient with diabetes mellitus in a Nigerian teaching hospital Hongkong J Psychiatri 2006 16 27 33

[B16] Soegondo S The Consensus of type 2diabetes mellitus management and prevention in Indonesia PB PERKENI 2006 12 15

[B17] Brown SA Craig I Hanis A community-based, culturally sensitive education and group-support intervention for Mexican Americans with NIDDM: A pilot study of efficacy The diabetes educator 1995 21 Suppl 3 203 210 7758387 10.1177/014572179502100307

[B18] Slevin K Cleator J Wilding J Frost G, Dornhost A, Moses R Obesity and diabetes Nutritional management of diabetes mellitus 2003 Wiley, England 112

[B19] Ramachandran A Snehalatha C Michael J, Barrie M, John M, Lenore Arab, Gibney , Kearney The Nutrition Society Textbook Series Public Health Nutrition 2004 [www.nutritiontexts.com]

[B20] Soegondo S Integrated diabetes mellitus management 2007 Balai Penerbit Fakultas Kedeokteran Universitas Indonesia, Jakarta

[B21] Pratiknya AW Basics of Research metodology in health and medicine 2000 PT Raja Grafindo Persada, Jakarta

[B22] Green LW Kreuter MW Health promotion planning: an educational and environmental approach 2000 2 Mayfield Publishing Company, California

[B23] Fountain S Assessment strategies for skills-based health education with focus on HIV prevention related issues. UNICEF education 2002

[B24] Azwar S Human attitude: theory and measurements 1995 2 Pustaka Pelajar, Yogyakarta Indonesia

